# Seven-Gene Signature and Immune Microenvironment as Determinants of Survival Rate in Colorectal Carcinoma

**DOI:** 10.32604/or.2026.077466

**Published:** 2026-10-28

**Authors:** Vincenzo Rallo, Matteo Massidda, Xiaofen Wen, Manila Deiana, Andrea Maschio, Jiaxin Shen, Maria Chiara Ninniri, Daniela Piras, Ciriaco Carru, Donatella Coradduzza, Maria Rosaria Muroni, Paolo Cossu-Rocca, Antonio Mario Scanu, Andrea Angius, Maria Rosaria De Miglio

**Affiliations:** 1 Institute of Genetic and Biomedical Research (IRGB), National Research Council (CNR), Monserrato, Italy; 2 Department of Biomedical Sciences, University of Sassari, Sassari, Italy; 3 Department of Medical Oncology, Cancer Hospital of Shantou University Medical College, Shantou, China; 4 Department of Hematology, The First Affiliated Hospital of Shantou University Medical College, Shantou, China; 5 Department of Medicine, Surgery and Pharmacy, University of Sassari, Sassari, Italy; 6 Complex Structure Epidemiology and Cancer Registry North Sardinia, ATS Sardinia, Sassari, Italy

**Keywords:** Colorectal cancer, transcriptome, immune microenvironment, hub genes, prognostic classifier

## Abstract

**Backgrounds:** Colorectal cancer (CRC) prognosis remains difficult due to molecular heterogeneity and interaction between tumor cells and the immune microenvironment. This study aimed to identify transcriptomic and immune-cell patterns associated with overall survival (OS) and to develop an integrated prognostic model to improve risk stratification. **Methods:** RNA-sequencing was performed on 131 primary CRC samples and matched normal tissues. Differentially expressed genes (DEGs) were identified and functionally characterized through gene ontology, Kyoto Encyclopedia of Genes and Genomes (KEGG) enrichment, and protein–protein interaction (PPI) network analysis. Immune-cell composition was estimated using CIBERSORTx deconvolution and evaluated for its association with OS. Prognostic DEGs were screened using univariate Cox regression and Least Absolute Shrinkage and Selection Operator (LASSO) analysis to construct a risk score. The model was validated in the Cancer Genome Atlas Database (TCGA)-COAD (colon cancer) and READ (rectal cancer) cohorts. A nomogram integrating molecular and clinicopathological variables were generated. **Results:** A total of 5589 DEGs were identified between CRC and normal tissues, enriched in pathways related to cell cycle, Tumor Protein P53 (TP53), WNT Family Member (WNT), Janus kinase/signal transducer and activator of transcription 3 (JAK/STAT), calcium signaling, metabolism, and immune regulation. PPI analysis highlighted ten upregulated hub genes involved in mitotic spindle formation and chromosomal stability. Immune infiltration analysys indicated that higher proportions of plasma cells (*p* = 6.9 × 10^−^^4^), naïve B cells (*p* = 0.019), resting CD4^+^ memory T cells (*p* = 0.02), M0 macrophages (*p* = 0.0077), and activated dendritic cells (*p* = 1.89 × 10^−^^5^) were associated with improved OS, whereas monocytes (*p* = 0.012), neutrophils (*p* = 0.041), activated mast cells (*p* = 0.0066), and M2 macrophages (*p* = 0.014) were linked to poorer survival. A seven-gene signature including aspartate beta-hydroxylase (ASPH), bradykinin receptor B1 (BDKRB1), calcium voltage-gated channel auxiliary subunit beta 1 (CACNB1), C-C motif chemokine receptor 8 (CCR8), cyclic nucleotide gated channel subunit alpha 3 (CNGA3), microtubule associated protein 1A (MAP1A) and oxytocin receptor (OXTR) stratified patients into high- and low-risk groups with significant OS differences. The model demonstrated strong predictive performance (AUC: 0.84 at 1 year) and was validated in TCGA cohorts. Multivariate analysis confirmed the risk score as an independent prognostic factor. The integrated nomogram accurately predicted 1-, 3-, and 5-year survival (C-index = 0.757; 95% CI 0.724–0.791). **Conclusions:** We developed and validated a seven-gene immune-related prognostic signature that, combined with clinicopathological parameters, provides a robust tool for individualized survival prediction and may guide precision management in CRC patients.

## 1 Introduction

Colorectal cancer (CRC) is the third most frequent cancer and the second leading cause of cancer death [1]. CRC incidence is expected to rise by 60% in 2030, leading to over 2.2 million new cases and 1.1 million deaths [2]. Twenty-five or thirty percent of patients present untreatable metastases at diagnosis [3]. Recurrence and drug resistance are major contributors to treatment failure. CRC’s molecular heterogeneity explains differences in outcomes and responses to therapy, complicating prognostic prediction [4]. The lack of reliable prognostic models and genetic markers results in late diagnoses and non-personalized treatments. Five-year survival at stages I and II is around 90%, but it falls to 10% at stage IV [5]. Demographic and clinic-pathological data can help predict survival and guide treatment choices [6]. While the American Joint Committee on Cancer (AJCC) staging system offers prognostic insights, treatment response and survival remain unpredictable [7].

In clinical practice, biomarkers like microsatellite instability (MSI), molecular subtypes (CMS), and tumor mutational burden (TMB) are used, while B-Raf Murine Sarcoma Viral Oncogene Homolog B (BRAF), Kirsten Rat Sarcoma Viral Proto-Oncogene (KRAS), and Neuroblastoma RAS Viral Oncogene Homolog (NRAS) mutations predict response to epidermal growth factor receptor (EGFR) inhibitors in metastatic CRC (mCRC). BRAF V600E mutation indicates a more aggressive course [8]. MSI-High (H) CRCs respond well to anti-programmed death-1 (PD1) therapy [9].

In recent years, several prognostic models have been developed to refine risk stratification in CRC. Assays such as ColoScape enable multiplex detection of recurrent mutations in key CRC genes [e.g., APC Regulator of Wnt Signaling Pathway (APC), KRAS, BRAF, Catenin Beta 1 (CTNNB1)] and show promising performance for early detection. The mutational rate in CRC patients was 100% in cell free DNA isolation (cfDNA), and 96.0% in formalin-fixed, paraffin-embedded (FFPE) samples. In patients with precancerous lesions, this assay has 62.5% of sensitivity and 95.2% of specificity with an area under curve (AUC) of ~0.79 [10]. However, their reliance on a restricted mutational panel limits their ability to capture the broader molecular heterogeneity of CRC.

Established biomarkers, including microsatellite instability (MSI) [11] and the Consensus Molecular Subtypes (CMS) [12], provide robust biological stratification but are based on categorical frameworks that do not fully reflect the dynamic interplay between tumor transcriptomics and the immune microenvironment. Similarly, Oncotype DX recurrence score, built on a seven-gene panel [biglycan (BGN), marker of proliferation Ki-67 (Ki67), MYB Proto-Oncogene Like 2 (MYBL2), growth arrest and DNA damage inducible beta (GADD45B), fibroblast activation protein alpha (FAP), inhibin subunit beta A (INHBA) and MYC Proto-Oncogene, BHLH Transcription Factor (C-MYC)], offers validated prognostic information in stage II/III CRC, yet its three-tier risk classification leaves a substantial proportion of patients in an intermediate-risk group with limited therapeutic guidance.

Emerging machine learning and multi-omics models [13,14] aim to integrate clinical and molecular data, but they often require large cohorts and may lack clinical interpretability. The multi-omics approach has been applied to CRC [15], and a recent meta-analysis assessed the clinical utility of various prognostic gene signatures [16]. Collectively, these limitations highlight the need for integrated prognostic strategies that move beyond tumor-cell genetics alone and incorporate immune and transcriptomic context to achieve more precise and clinically actionable outcome prediction.

Cell composition in tumor tissues has highlighted specific targets and drugs that could improve patient outcomes [17]. This perspective promotes a shift from a tumor-only approach to one involving the tumor microenvironment (TME), fostering precision therapy [18].

In this study, we aimed to investigate transcriptomic differences between primary colorectal cancer (CRC) and matched normal colorectal tissues (NCT) using RNA sequencing and integrative bioinformatic approaches, including Gene Ontology enrichment and protein–protein interaction (PPI) network analysis. We also sought to characterize tumor microenvironment immune-cell populations through immune deconvolution analysis and explore their association with patient survival. The specific objective of this study was to develop and validate a gene-based prognostic model for colorectal cancer and to integrate it with clinicopathological variables in a predictive nomogram, with the goal of improving individualized survival prediction for CRC patients.

## 2 Methods

### 2.1 Subjects and Generation of Transcriptomic Data

Study population comprised anonymized patients consecutively diagnosed with CRC and treated by surgical resection in the Surgery Unit II of Sassari University between 2018 and 2022. All cases achieved a final diagnosis of CRC according to World Health Organization criteria [19]. Patients provided written consent approved by the Azienda Ospedaliera Universitaria Cagliari Bioethics Committee (number PG/2020/19737 dated 25/11/2020). We structured a database with clinic-pathological features at diagnosis and follow-up.

Our cohort consisted of 135 fresh-frozen paired samples of primary CRC and NCT, taken about 10 cm from primary tumor. RNA extraction and quality assessment were performed as previously described [20]. RNA libraries were generated using Illumina^®^ Stranded Total RNA Prep (Cat. No. 20040525, Illumina, San Diego, CA, USA), and each was sequenced on an Illumina NovaSeq 6000 (Illumina, San Diego, CA, USA) with S4 Reagent Kit v1.5 (Cat. No. 20028312, Illumina, San Diego, CA, USA), in paired-end mode (2 × 100 bp).

### 2.2 Next Generation Sequencing Data Processing

Bioinformatic analysis utilized an in-house workflow based on the Snakemake framework [21], minimizing workload and management bias. Sequence quality control was performed using FastQC [22], MultiQC [23] and Cutadapt/TrimGalore [24]. Reads were aligned to the reference genome (build GRCh38/hg38) using STAR2 [25]. Differentially expressed genes DEGs were identified by DESeq2 R package version 1.38.3 [26], which normalizes for sequencing depth and RNA composition. Unsupervised hierarchical clustering was performed using Pearson’s correlation. Principal component analysis (PCA) was performed with a DESeq2 built-in function. A Volcano plot with DEGs was generated using ggplot2 R package 3.5.0. Comparisons of CRC vs. NCT were conducted to remove intraindividual variation, with cut-off parameters of *p* < 0.05 and |Log2 (Fold Change FC)| ≥ 0.58 [27]. Benjamini-Hochberg method adjusted DEGs’ setting false discovery rate (FDR) < 0.05 the significant threshold.

### 2.3 Functional Enrichment Analysis

Gene set enrichment analysis (GSEA) between CRC and NCT was conducted using the clusterProfiler R package 4.4.4 [28], considering terms with adjusted *p* < 0.05 and FDR < 0.25 as significant.

### 2.4 Protein-Protein Interaction (PPI) Network Analysis

The protein–protein interaction (PPI) network of DEGs was created using search tool for the retrieval of interacting genes/proteins (STRING) (https://www.string-db.org/), with a confidence score threshold of >0.7. The resulting network was visualized in Cytoscape software (Version 3.8.0, Cytoscape Consortium, San Diego, CA, USA), and top hub genes (HG) were identified using the MCC algorithm from CytoHubba (https://apps.cytoscape.org/apps/cytohubba).

### 2.5 Deconvolution Analysis

Deconvolution analysis highlighted differences in immune cell subtype composition between CRC and NCT. We integrated transcriptome data with a leukocyte gene signature matrix (LM22) comprising 22 immune cell types, characterized by 547 gene markers. Cell subtype abundances were estimated with cell-type identification by estimating relative subsets of RNA transcripts (CIBERSORTx; https://cibersortx.stanford.edu/), which infers cell type proportions from a single cell reference signature [29]. We set the following parameters: permutations = 1000, B-mode batch correction enabled, quantile normalization disabled, and relative run mode. A quality check was performed by selecting significant cell fractions (*p* value < 0.05). The Wilcoxon signed rank test was conducted using the wilcox_test function from the rstatix R package 0.7.2 to discern variations in representation of cell types. Statistical significance was adjusted with Bonferroni. The effect size was computed for each cell type using the wilcox_effsize function from rstatix [https://CRAN.R-project.org/package=rstatix]. For each cell type, CRCs were grouped into low and high fraction with optimal cutpoint computed with the surv_cutpoint function of the R package survminer 0.5.0, which applies maximally selected rank statistics from the maxstat package to identify the cutpoint with the strongest association with survival [https://CRAN.T-project.org/web/packages/survminer/index.html, https://cran.r-project.org/web/packages/maxstat/index.html]. Kaplan-Meier analysis was performed to investigate immune cell infiltration’s prognostic value in CRC tissues using survival R package3.5-5 [https://CRAN.R-project.org/package=survival].

### 2.6 Construction and Validation of a Prognostic Gene Signature

DEGs related to OS in CRC (*p* < 0.05) were identified using univariate Cox regression analysis with the coxph function of the R package survival 3.5-5. The Least Absolute Shrinkage and Selection Operator (LASSO) analysis was applied for variable selection to build an OS prediction model using the RLassoCox R package [29], based on the glmnet R package 4.1-8. RLassoCox integrates gene interaction from a Kyoto Encyclopedia of Genes and Genomes (KEGG) Network [30] into the LASSO-Cox model for accurate survival prediction. The penalty regularization parameter λ was determined via the cross-validation with an n-fold value equal to 10. The λ value was finalized by using lambda.min, which is the value of lambda giving minimum mean cross-validated error (λ = 0.4493671). 

Nine genes were identified via LASSO, and stepwise model selection using Akaike Information Criterion (MASS R package 7.3-58.3) reduced them to seven [https://CRAN.R-project.org/web/packages/MASS/index.html]. The risk score was calculated with the predict.coxph function of the R package survival, setting the parameters ‘type = lp’ and ‘reference = zero’, using the formula “RiskScore = (expr gene1 × β1) + (expr gene2 × β2) + (expr genen × βn)”, where “β” represents the coefficient of each gene and “expr” represents their expression level. Patients were divided into low- and high-risk groups based on the optimal cutpoint. Receiver Operating Characteristic analysis (ROC) was done with the timeROC R package [https://CRAN.R-project.org/web/packages/timeROC/index.html]. Relationships between risk scores and clinic-pathological data were assessed with the Kruskal-Wallis Test. Stratified survival analysis was performed to verify risk score robustness. Validation was conducted using the Cancer Genome Atlas Database (TCGA)-COAD (colon cancer) and TCGA-READ (rectal cancer) (https://portal.gdc.cancer.gov) cohorts, using RNASeq data derived from 642 CRC samples (COAD = 478, READ = 164). Functional enrichment of the seven genes signature was conducted via string-db.org.

### 2.7 Distribution and Correlation between Risk Score, Immune Infiltration and Clinical-Pathological Features

Differences in level of immune cells infiltration between risk score groups were evaluated using Wilcoxon rank sum test. Spearman’s correlation analyzed risk score and immune cells. The Kruskal-Walli’s test, followed by Dunn’s multiple comparison test, was used to compare clinical pathological features based on risk score. To characterize the tumor immune microenvironment (TIME), we performed an extensive association analysis between 22 immune cell populations (inferred using CIBERSORTx) and key clinicopathological variables, including age, tumor site, pT, pN, pM, stage, grade, lymphovascular invasion (LVI), and perineural invasion (PNI). Group comparisons were conducted using the Kruskal–Wallis test, followed by Dunn’s post-hoc test with Bonferroni correction for multiple comparisons.

### 2.8 Independent Prognostic Factors Analysis and Nomogram Prediction Model Construction

Uni- and multivariate Cox regression analyses identified independent prognostic indicators. A nomogram was developed to predict CRC prognosis based on the risk scores and clinic-pathological characteristics, enabling calculation of 1-year, 3-year, and 5-year survival probabilities for each patient. The “rms” R package 6.8-0 was utilized for nomogram and calibration plots [https://CRAN.R-project.org/package=rms]. Predictive accuracy was evaluated using time-dependent ROC curves. Prediction accuracy was further evaluated using time-dependent Brier scores and the integrated Brier score (IBS) with the survival (v3.3-1) and pec R packages, where lower values indicate better predictive performance. Clinical utility was assessed using decision curve analysis (DCA). The Cox model was fitted using the survival package, and DCA was performed using the stdca.R script to estimate net benefit across threshold probabilities.

### 2.9 Statistical Analysis

All statistical analyses were performed using R software (version 4.2.3). Continuous variables were summarized as mean ± standard deviation (SD) or median (interquartile range, IQR), according to data distribution, and categorical variables as frequencies and percentages. Associations with overall survival (OS) were evaluated using uni- and multivariable Cox proportional hazards models, reporting hazard ratios (HRs) with 95% confidence intervals (CIs). Independent prognostic factors were integrated into a nomogram to estimate 1-, 3-, and 5-year OS probabilities. Model performance was assessed using the concordance index (C-index), time-dependent ROC curves, calibration plots, Brier score, and decision curve analysis (DCA). Two-sided *p* values < 0.05 were considered statistically significant.

## 3 Results

### 3.1 Transcriptome Sequencing and Mapping

Fig. 1 shows the study’s flow diagram, detailing the research process. 135 paired tissues from primary CRC and distant NCT underwent high-throughput transcriptome sequencing (~75 million 101-bp paired-end reads per sample). After histological review, four non-CRC samples were excluded (two low-grade polyps and two intestinal lymphomas). RNA-Seq data for 131 CRC and matched NCT samples were aligned to the UCSC human genome GRCh38/hg38. Clinicopathological features at diagnosis are in Table S1.

**Figure 1:**
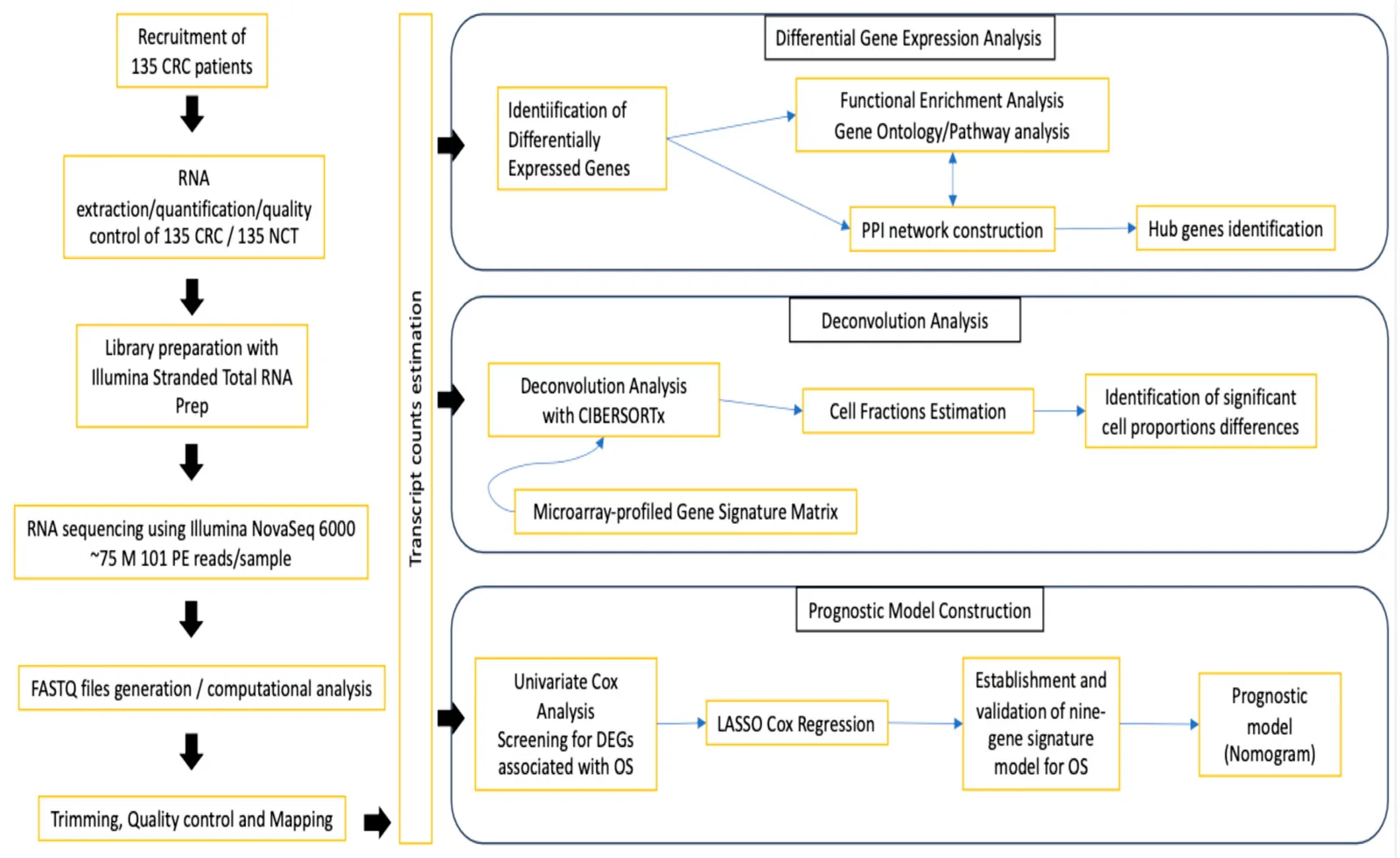
Schematic representation of the sequential steps of the research processes. Note: CRC, colorectal cancer; NCT, normal colon tissue; PPI, protein-protein interaction; CIBERSORTx, cell-type identification by estimating relative subsets of RNA transcripts; DEGs, differentially expressed genes; OS, overall survival; LASSO, least absolute shrinkage and selector operation.

### 3.2 Gene Expression Profiling between Colorectal Cancer and Matched Normal Colon Tissues

About 36,500 genes were identified. Between CRC and NCT, 5589 DEGs were found: 2036 overexpressed and 3553 downregulated in CRC (Table S2). Unsupervised clustering of the top 1% variable genes (n = 366) showed distinct DEG clusters between CRC and NCT (Fig. 2A). Volcano plot visualized gene expression levels (Fig. 2B), and PCA demonstrated distinct clustering of CRC and NCT datasets (Fig. 2C).

Functional enrichment analysis explored the role of 5589 DEGs. Enriched DEGs in KEGG pathways linked to cancer biology are shown in Fig. 2D. Other relevant pathways include cytokine-cytokine receptor interaction, toll-like receptor, neuroactive ligand-receptor interaction, calcium signaling, DNA replication, spliceosome, p53, JAK/STAT, and WNT pathways (Table S3). The top five biological processes (BP), cellular components (CC), and molecular functions (MF) are in Fig. 2E–G. Noteworthy BPs for CRC include chromosome segregation, DNA metabolic processing, cell cycle regulation, p53-mediated signal transduction, microtubule cytoskeleton organization, mucosal immune response, corticosteroid response, molecular mediator production, immune regulation, inflammatory response, calcium ion homeostasis, and xenobiotic metabolic processing. DEG enrichment in BP, MF, and CC is detailed in Table S4.

**Figure 2:**
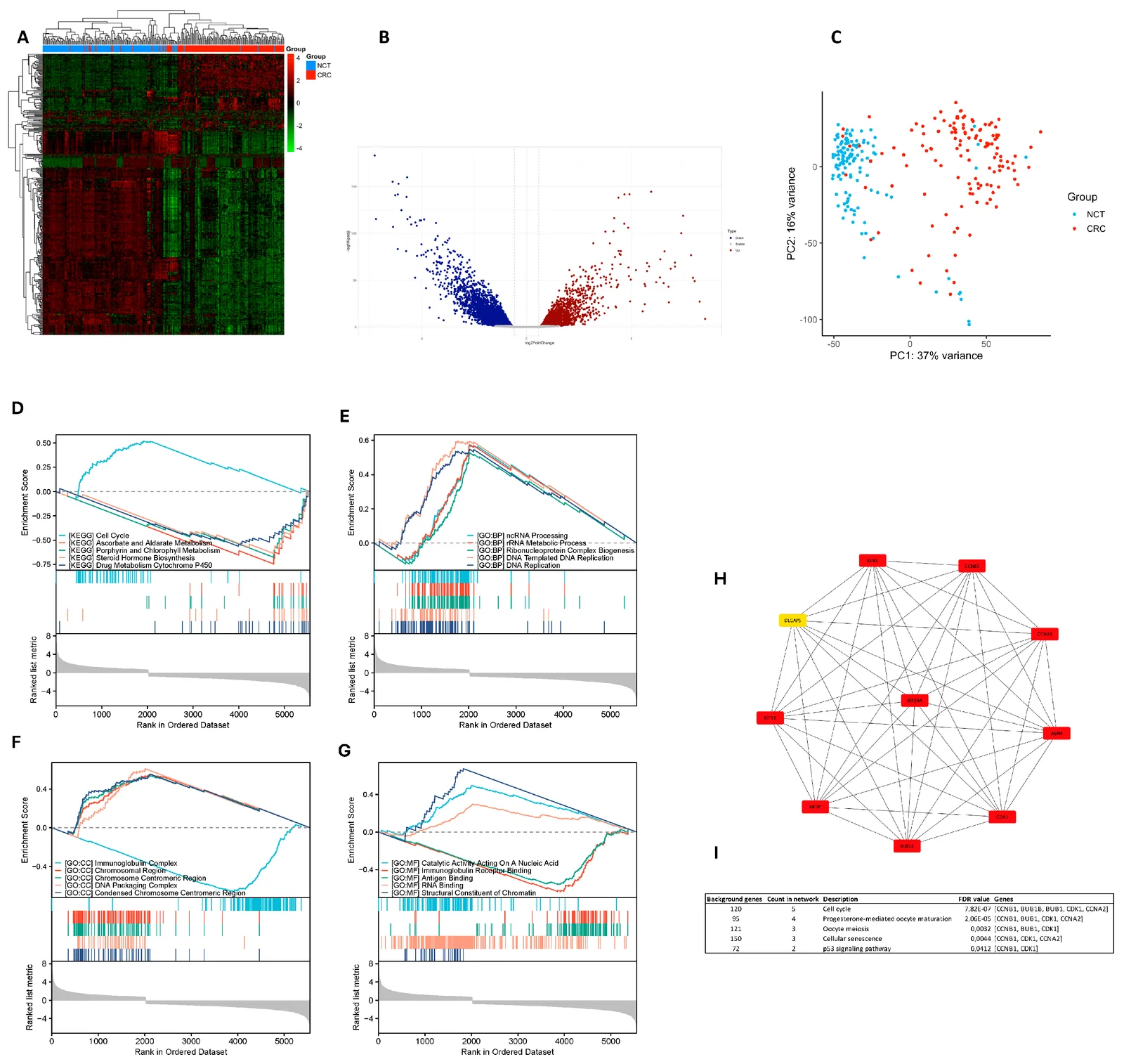
Heatmap, volcano plot and principal component analysis (PCA) of differentially expressed genes (DEGs) between colorectal cancer (CRC) and normal colon tissue (NCT). (**A**) Unsupervised hierarchical clustering analysis of the 366 more significant out of 5589 DEGs. CRC and NCT groups were represented in the red and blue color, respectively; (**B**) Transcriptional expression levels of all genes were visualized by volcano plot: blue dots; downregulated trends, red dots; upregulated trends and gray dots; genes without significant differences; (**C**) PCA analysis plot based on transcriptome data of two groups; red; tumor tissue and blue; normal tissue; (**D**) Gene set enrichment analysis (GSEA) of DEGs strongly related to CRC. Enrichment plot for the five gene sets (KEGG pathways); (**E**) Enrichment plot for the five gene sets (Gene Ontology (GO) biological processes); (**F**) Enrichment plot for the five gene sets (GO cellular components); (**G**) Enrichment plot for the five gene sets (GO molecular functions). Pictures were created using MSigDB; (**H**) Protein-protein interaction (PPI) network of DEGs in CRC. Top 10 hub genes screened from the PPI network using the MCC algorithm of the CytoHubba clustering plugin in Cytoscape (https://cytoscape.org/); the red nodes have higher MCC and yellow nodes a lower MCC; (**I**) KEGG pathways involving the hub genes. Note: CDK1, cyclin dependent kinase 1; BUB1B, mitotic checkpoint serine/threonine kinase B; BUB1, mitotic checkpoint serine/threonine kinase; ASPM, assembly factor for spindle microtubules; KIF11, kinesin family member 11¸KIF20A, kinesin family member 20A; CCNB1, cyclin B1; KIF2C, kinesin family member 2C; CCNA2, cyclin A2; DLGAP5, DLG associated protein 5.

### 3.3 Protein-Protein Interaction (PPI) Analysis

A PPI network examined functional protein interactions among DEGs. A total of 4314 nodes and 18,431 edges were mapped, with significant PPI enrichment (*p* < 1.0e−16). The network revealed 10 key hub genes (HGs; Fig. 2H), all upregulated in CRC vs. NCT [cyclin dependent kinase 1 (CDK1), mitotic checkpoint serine/threonine kinase B (BUB1B), mitotic checkpoint serine/threonine kinase (BUB1), assembly factor for spindle microtubules (ASPM), kinesin family member 11 (KIF11), kinesin family member 20A (KIF20A), cyclin B1 (CCNB1), kinesin family member 2C (KIF2C), cyclin A2 (CCNA2), and DLG associated protein 5 (DLGAP5)]. KEGG pathway analysis showed HG enrichment in cell cycle, senescence, and TP53 signaling (Fig. 2I).

### 3.4 Deconvolution Analysis

The deconvolution analysis identified distinct immune cell populations in the CRC microenvironment. Macrophages M0 (*p* = 7.33e−17), M1 (*p* = 4.18e−05), activated mast cells (*p* = 1.86e−13), resting Natural Killer (NK) cells (*p* = 7.744e−12), activated dendritic cells (*p* = 3.06e−07), activated memory CD4 T cells (*p* = 1.13e−06), eosinophils (*p* = 1.83e−03), neutrophils (*p* = 4.71e−04), and naïve CD4 T cells (*p* = 2.33e−04) were more abundant in CRC than NCT. In contrast, resting mast cells (*p* = 3.48e−18), macrophages M2 (*p* = 1.85e−10), activated NK cells (*p* = 2.64e−06), plasma cells (*p* = 6.36e−08), CD8 T cells (*p* = 3.06e−04), and memory B cells (*p* = 2.51e−04) were prevalent in NCT (Fig. 3A).

Significant differences (*p* adj < 0.001) were seen in activated memory CD4 T cells, NK cells (resting and activated), macrophages (M0 and M2), plasma cells, and mast cells (resting and activated), with notable effect sizes. Dendritic cells (activated), T-cells (CD4 naïve and CD8), memory B cells, eosinophils, macrophages (M1), and neutrophils also showed significant differences with moderate effect sizes (Table S5).

The estimated proportions of immune cell types were linked to OS (Fig. 3B). High fractions of plasma cells (*p* = 6.9e−04), naïve B cells (*p* = 0.019), resting memory CD4 T cells (*p* = 0.02), macrophages M0 (*p* = 0.0077), and activated dendritic cells (*p* = 1.89e−05) were related to better prognosis. Conversely, higher levels of monocytes (*p* = 0.012), M2 macrophages (*p* = 0.0014), neutrophils (*p* = 0.041), and activated mast cells (*p* = 0.0066) were associated with lower survival rates.

**Figure 3:**
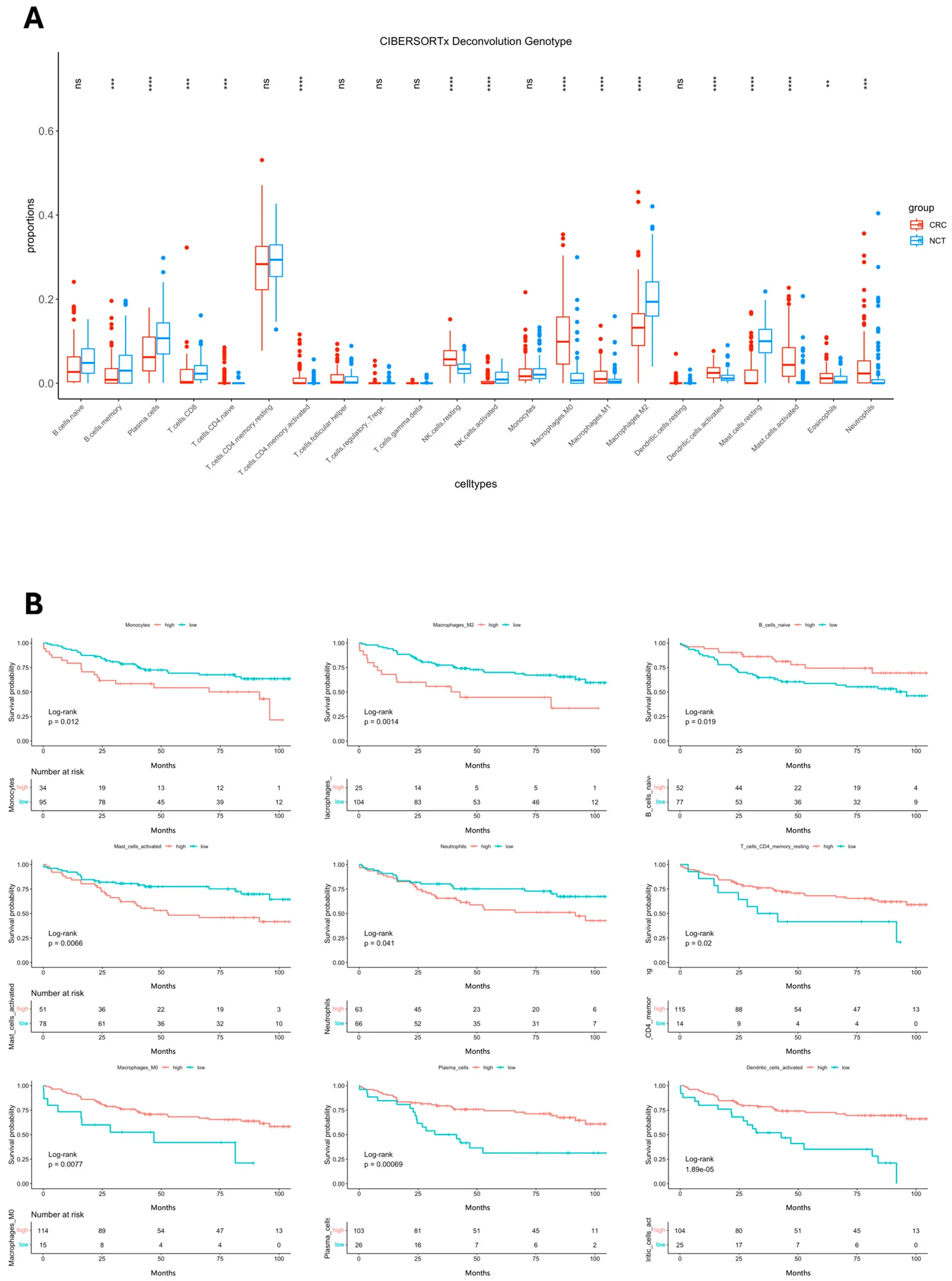
Deconvolution of cell types and prognostic values of immune cells. (**A**) The boxplots illustrate the distribution of various cell types in colorectal cancer and normal colon tissue, highlighting the differential representation and providing indications of alterations in cell composition between the two groups. Significant differences between the two groups: *p* < 0.01 = **; *p* < 0.001 = ***; *p* < 0.0001 = ****; ns: not significant. (**B**) Prognostic values of immune cells infiltration in tissues of colorectal cancer patients assessed by the Kaplan-Meier method. Survival curves were graphically represented using the survminer R package. On the horizontal axis, the unit of time is stated in months.

### 3.5 Construction and Validation of DEGs-Related Prognostic Signature

Univariate Cox regression analysis of 5589 DEGs identified 571 genes with strong prognostic significance (Table S6). To prevent overfitting, LASSO-Cox regression was used, narrowing the genes down to nine using the minimum lambda value (λ) (Fig. 4A). Stepwise selection yielded a final prognostic signature of seven genes. The risk score formula was: Risk score = (4.72e−05 ∗ aspartate beta-hydroxylase ASPH) + (1.82e−03 ∗ bradykinin receptor B1, BDKRB1) + (1.99e−03 ∗ calcium voltage-gated channel auxiliary subunit beta 1, CACNB1) + (−2.09e−02 ∗ C-C motif chemokine receptor 8, CCR8) + (9.33e−03 ∗ cyclic nucleotide gated channel subunit alpha 3, CNGA3) + (1.95e−04 ∗ microtubule associated protein 1A, MAP1A) + (4.89e−03 ∗ oxytocin receptor, OXTR).

To validate the model, patients were split into high-risk (n = 28) and low-risk (n = 103) groups based on an optimal risk score cut point (1.52). Higher scores correlated with a worse prognosis (Fig. 4B). Kaplan-Meier curves confirmed that high-risk patients had worse outcomes (*p* < 0.05) (Fig. 4C). Time-dependent ROC analysis showed an OS prediction accuracy of 0.84 at 1 year, 0.76 at 3 years, and 0.75 at 5 years (Fig. 4D), indicating the model’s robustness.

**Figure 4:**
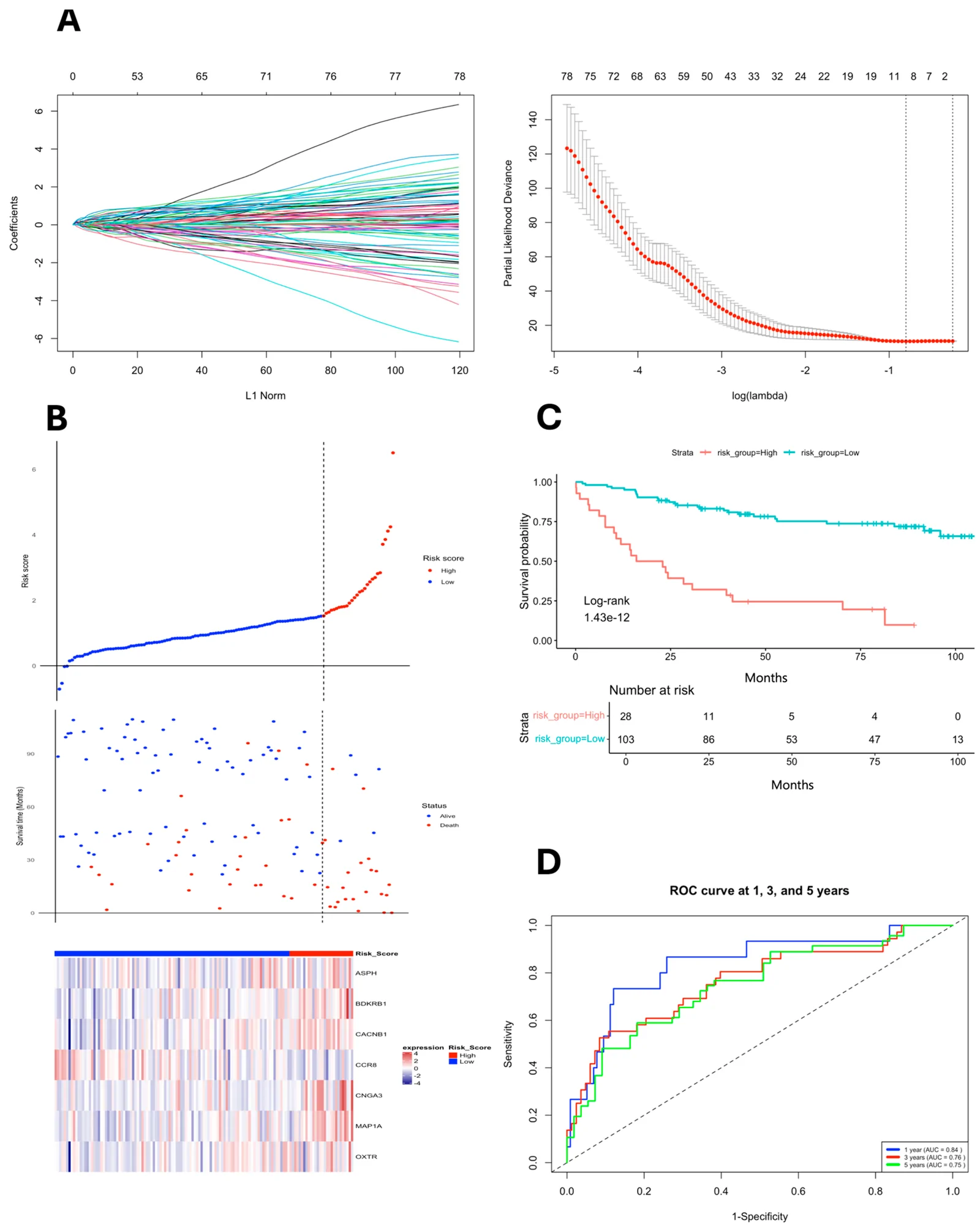
Construction of the risk score model using the least absolute shrinkage and selector operation (LASSO) logistic regression analysis and 10-fold cross-validation. (**A**) LASSO coefficient profiles of the genes associated with overall survival in colorectal cancer; deviance of partial likelihood plotted against log (Lambda): the dashed vertical line indicates the lambda value with the smallest error and the largest lambda value where the deviance is within one SE from the minimum. (**B**) Risk score, survival status and risk heat map analysis for patients in the high- and low-risk groups. (**C**) Kaplan Meier curves for overall survival of patients in the high- and low-risk groups. (**D**) Receiver Operating Characteristic curve analysis (ROC) for the feasibility of a seven-gene prognostic model.

Validation in TCGA-COAD and TCGA-READ cohorts (Fig. 5A,B) demonstrated good specificity and sensitivity. The seven-gene signature was enriched in KEGG calcium signaling pathways, Wiki pathways (Peptide G-protein-coupled receptors (GPCRs) and GPCRs class A rhodopsin-like), and BPs related to positive regulation of cytosolic calcium ion concentration (Fig. 5C).

**Figure 5:**
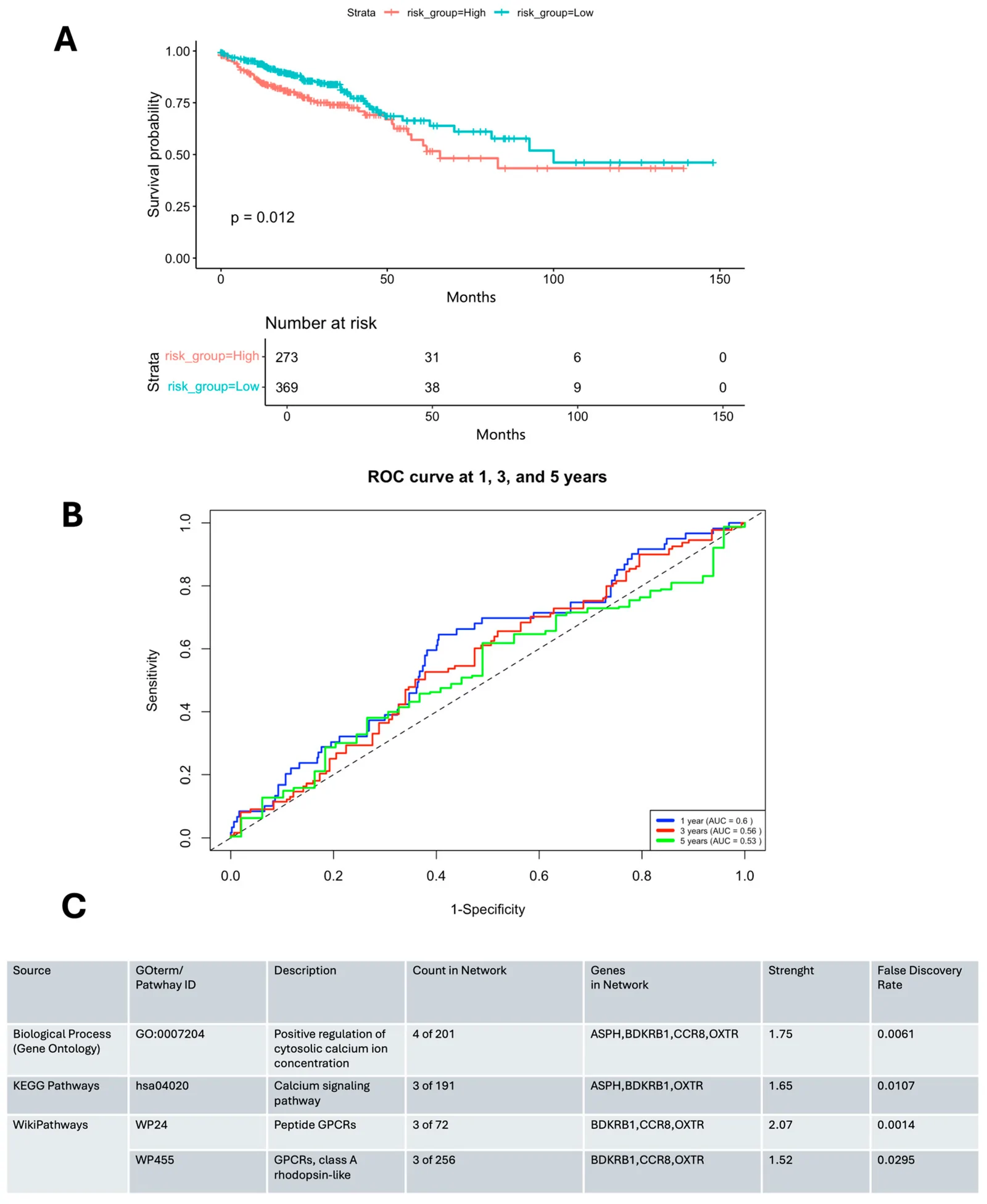
Validation of the differential expressed genes-related prognostic signature in The Cancer Genome Atlas Database (TCGA)-COAD (colon cancer)/READ (rectal cancer) cohort. (**A**) Kaplan Meier curves for the overall survival of patients from the TCGA-COAD/READ. cohort. (**B**) Prognostic performance of the risk score in TCGA-COAD/READ cohort, verified by area under curve (AUC) of time-dependent receiver operating characteristic (ROC) curves. (**C**) Enrichment findings involving the seven-gene signature. Note: ASPH, aspartate beta-hydroxylase; BDKRB1, bradykinin receptor B1; CCR8, C-C motif chemokine receptor 8; OXTR, oxytocin receptor.

### 3.6 Distribution and Correlation between Risk Score, Immune Infiltration and Clinical-Pathological Features

We analyzed immune cell distribution in high- and low-risk groups, finding increased T cells CD4 memory resting, macrophages M0, and activated dendritic cells in the low-risk group, while monocytes were higher in the high-risk group (*p* < 0.05) (Fig. 6A). Risk score positively correlated with neutrophils (r = 0.213, *p* < 0.05) and monocytes (r = 0.189, *p* < 0.05), and negatively with T cells CD4 memory resting (r = −0.268, *p* < 0.01), macrophages M1 (r = −0.203, *p* < 0.05), and both resting and activated dendritic cells (r = −0.202, *p* < 0.05; r = −0.185, *p* < 0.05) (Fig. 6B).

Risk scores were stratified by clinical features, showing higher scores in patients with advanced Tumor–Node–Metastasis (TNM), stage IV, and LVI, but no significant differences for age, gender, site, grade, tumor infiltrating lymphocytes (TILs), or PNI (Fig. S1, Table S7). Higher risk scores predicted worse survival outcomes across subgroups based on age, sex, tumor location, grade, pT, pN, pM, stage, TILs, and presence/absence of LVI or PNI (*p* < 0.05).

The analysis of the relationship between immune profile and clinical features revealed a robust correlation between TILs score and the infiltration of pro-inflammatory populations, specifically M1 Macrophages (*p* adj < 0.001), Tregs (*p* adj = 0.004) and CD8^+^ T cells (*p* adj = 0.042), CD8^+^ T cells are increased in stage IV vs. III (*p* adj = 0.004) confirming that our deconvolution analysis effectively captures the transition toward an “immunologically hot” phenotype. A negative correlation has been identified between stage IV vs. III and B cell memory (*p* adj = 0.001), while mast cell activated are positively associated with IV vs. II (*p* adj = 0.005). We observed a significant depletion of memory B cells in patients with metastatic disease (pM1 vs. pM0; *p* = 0.004) and T cell CD4 memory resting (*p* adj = 0.004 and *p* adj = 0.002), memory B cells are decreased also in advanced stages (stage IV vs. III; *p* adj = 0.001), suggesting a progressive impairment of the adaptive humoral response during CRC systemic dissemination. Interestingly, the tumors involving the left side of the colon are enriched of mast cell activated (*p* adj = 0.005). All results are described in Figs. S2 and S3 that contains the boxplots of all significant associations and in Table S8 reporting all nominal and adjusted *p*-values.

**Figure 6:**
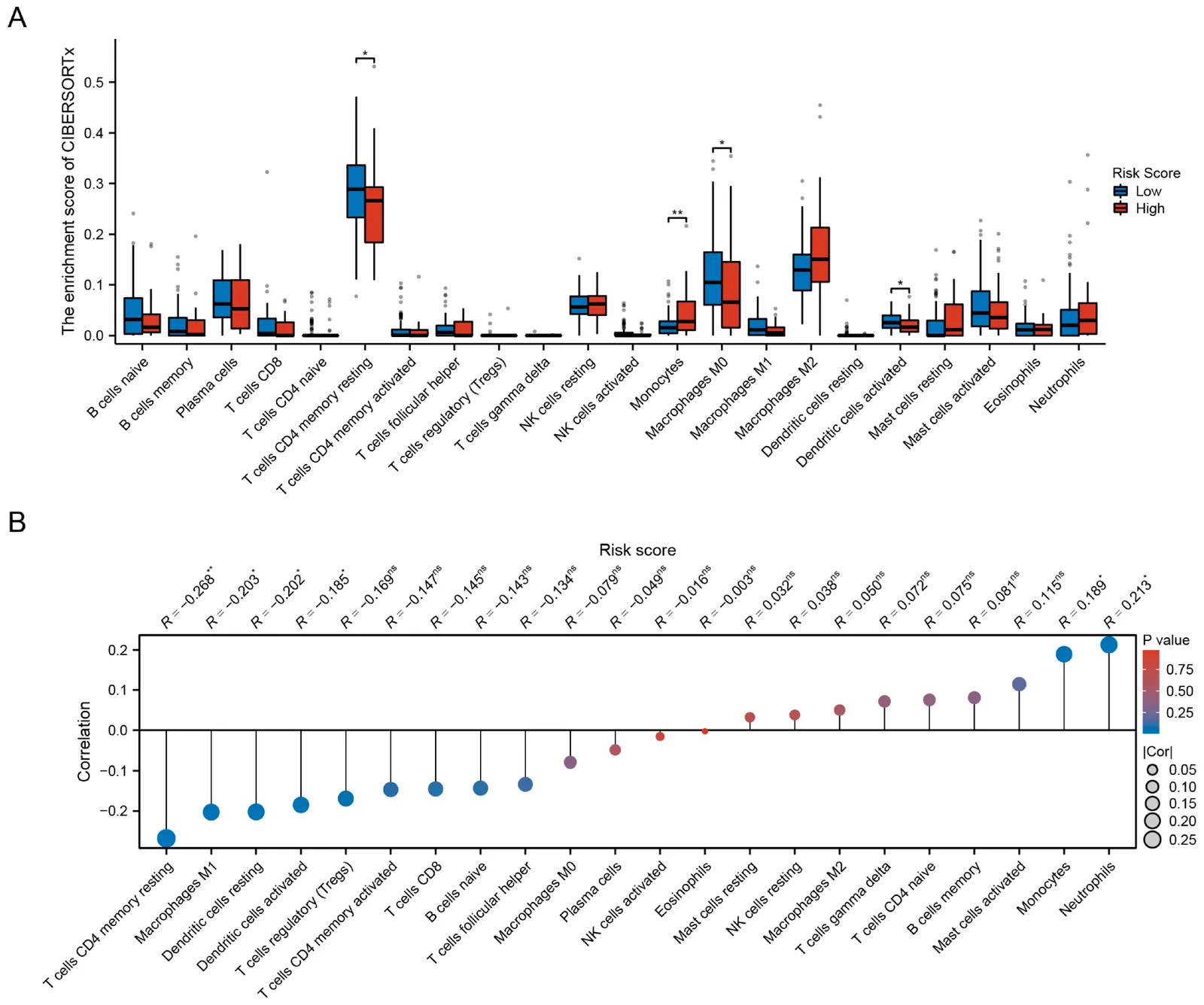
Correlation between risk score and immune cells infiltration in colorectal cancer. (**A**) Comparison of immune cells infiltration levels between the high- and low-risk score groups. (**B**) Correlation between risk score and relative abundance of immune cells. Dot size is matched to the absolute Spearman’s correlation coefficient values. (*p* < 0.05 = *; *p* < 0.01 = **; ns: not significant).

### 3.7 Independent Prognostic Factors Analysis and Nomogram Prediction Model Construction

Univariate Cox regression identified several risk factors associated with OS, including pN2 (HR = 2.681, *p* = 0.003), pM1 (HR = 4.033, *p* < 0.001), LVI (HR = 2.237, *p* = 0.01), PNI (HR = 2.680, *p* = 0.004), and high-risk group (HR = 6.262, *p* < 0.001) (Fig. 7A). Multivariate analysis confirmed that pM1 (HR = 3.670, *p* < 0.001) and high-risk group (HR = 6.353, *p* < 0.001) were independent prognostic predictors.

A nomogram was then developed to predict 1-, 3-, and 5-year survival (Fig. 7B), demonstrating good discriminative ability (C-index: 0.757, 95% CI: 0.724–0.791) and favorable predictive accuracy as assessed by Brier score (Fig. 7C). Time-dependent ROC curves showed strong diagnostic performance, with AUCs for pM and risk score of 0.761 and 0.815 (1 year), 0.727 and 0.711 (3 years), and 0.684 and 0.691 (5 years) (Fig. 7D). Calibration curves confirmed excellent agreement between predicted and observed survival probabilities (Fig. 7E).

Decision curve analysis (DCA) indicated that the nomogram provided a higher net clinical benefit across a wide range of threshold probabilities compared with individual factors (pM and risk score), supporting its potential utility for clinical decision-making (Fig. 7F).

**Figure 7:**
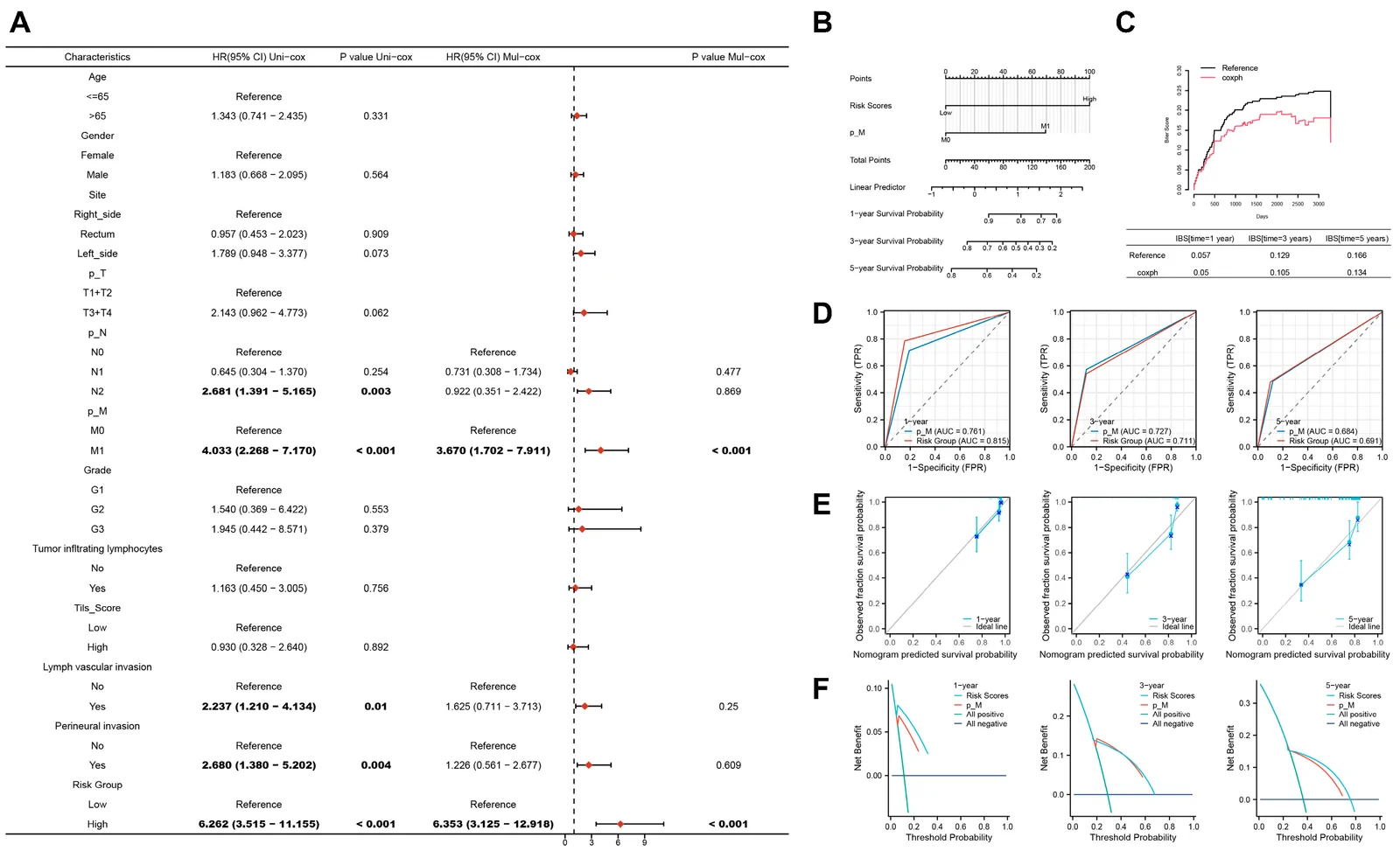
Construction of a nomogram predicting the overall survival (OS) of colorectal cancer (CRC) patients. (**A**) Forest map based on uni- and multivariate Cox analysis for OS. (**B**) Nomogram for predicting the probability of 1-, 3- and 5-year OS in CRC patients. (**C**) Prediction error curves showing the integrated Brier score (IBS) of the nomogram, with corresponding 1-, 3-, and 5-year Brier score values presented in the table. (**D**) Time-dependent survival ROC curve analysis. (**E**) Calibration plots validating the efficiency of nomograms for OS. (**F**) Prediction error curves showing the integrated Brier score (IBS) of the nomogram, with corresponding 1-, 3-, and 5-year Brier score values presented in the table. Note: HR, hazard ratio; Uni-Cox, univariate-Cox analysis; Mul-Cox, multivariate-Cox analysis; TILs, tumor infiltrating lymphocytes; AUC, area under curve.

## 4 Discussion

Despite advancements in understanding CRC biology, heterogenous treatments responses and frequent recurrence remain major clinical challenges. High throughput sequencing has improved molecular classification and the prognostic signatures, although multi-omics analyses are often limited by the availability of well-characterized tumor samples [4].

We compared 131 primary CRC samples with matched NCT, identifying 5589 DEGs. The use of paired samples for each patient represents a key strength, allowing a more accurate comparison than analyses based solely on public datasets. Most DEGs were enriched in pathways central to tumorigenesis, including the cell cycle, calcium signaling, TP53, WNT, and JAK/STAT pathways, as well as metabolic processes (carbohydrate, lipid, retinol, and drug metabolism via cytochrome P450), and mechanisms related to DNA replication, repair, and transcription. We also observed enrichment in pathways regulating the TIME, consistent with our previous transcriptomic findings [20].

The PPI network identified ten HGs highly connected, all upregulated in CRC. The literature supports their role to CRC pathogenesis, particularly in cell cycle regulation. Except for CCNA2, these HGs are mitotic regulators involved in spindle apparatus formation (SAF) whose deregulation contributes to chromosomal instability (CIN), a hallmark in CRC.

ASPM is involved in cell cycle progression, DNA double-strand break repair, and stemness regulation. It is overexpressed in advanced CRC, and its silencing reduces proliferation and metastasis, partly through β-catenin-dependent mechanisms [31]. 

DLGAP5, also overexpressed in CRC, correlates with advanced tumor stages and its downregulation limits metastatic potential [32,33]. 

CDK1, CCNB1, and CCNA2 regulate the G1/S and G2/M transitions. Their overexpression carries prognostic value and highlights these molecules as potential therapeutic targets [34]. CDK1 overexpression is linked to poor prognosis and with apoptosis resistance in BRAFV600E CRC, while its inhibition increases sensitivity to 5-fluorouracil. Combined targeting of CDK1 and MEK/ERK inhibition, presents a potential therapeutic strategy [35].

Kinesins (KIFs) essential for spindle dynamics and chromosome segregation, influence proliferation, invasion, and immune responses in CRC cells. KIF20A is associated with poor OS, and its suppression enhances apoptosis induced by fluorouracil and oxaliplatin, partly through modulation of JAK2 and signal transducer and activator of transcription 3 (STAT3) phosphorylation [36]. KIF2C predicts lymph node metastasis and poor prognosis in CRC [37,38], while KIF11 correlates with advanced stage and vessel invasion, its silencing increases DNA damage and oxaliplatin sensitivity [39].

BUB1B and BUB1 are key components for spindle checkpoint function. BUB1B is linked aneuploidy and genomic instability, and its silencing arrests cell proliferation through JNK/c-JUN pathway inhibition [40]. BUB1, overexpressed in aggressive cancer subtypes, correlates with tumor stage, size, invasiveness, hypoxia, and stemness [41].

Overall, the identified HGs may serve as prognostic biomarkers and potential therapeutic targets in CRC, particularly in relation to stemness regulation and mitotic spindle assembly.

We applied deconvolution methods to compare the immune landscape in CRC compared to NCT, revealing significant differences in immune cell composition and their association with OS.

A central finding is that a higher proportion of plasma cells in NCT vs. CRC correlates with improved OS. Although previous studies have reported plasma cell infiltration in CRC using cytofluorometry and immunohistochemistry, only a few studies have linked their presence to favorable prognosis [42,43]. Our results support their antitumor role, likely mediated through cytokines and antibody production cellular cytotoxicity, phagocytosis, complement activation, and enhanced antigen presentation [44,45].

Higher proportion of naïve B cells and resting CD4^+^ T memory cells are associated with improved OS. Naïve B cells decrease with advancing tumor stage and their infiltration—particularly within the CD73^+^ B-cell subset—has been linked to improved survival [46]. Naïve B cells regulate interferon gamma (IFN-γ) and tumor necrosis factor alfa (TNF-α) by T cells. These cytokines are critical in early tumor control and in sustaining antitumor immunity in advanced CRC [47]. Resting CD4^+^ T memory cells were similarly associated with reducing relapse risk and better response to preoperative radiotherapy reinforcing their protective role [48].

We observed a significant increase in activated dendritic cells in CRC, which positively correlated with OS [49]. Tumor-infiltrating dendritic cells were negatively correlated with lymph node metastasis, tumor size, stage, and survival rates. Their dysfunction in CRC is partly driven by tumor-derived IL-10 and TGF-β, which impair antigen presentation and T-cell priming [50]. The presence of mature dendritic cells in MSI-H tumors may contribute to enhanced immunotherapy responsiveness [51].

High levels of monocytes, M2 macrophages, mast cells, and neutrophils are associated with worse survival. Tumor hybrid cells (THCs), derived from fusion between tumor cells and monocytes/macrophages, promote metastasis and immune evasion, partly through sialic acid binding Ig like lectin 5 (SIGLEC5) expression [52]. Monocytes recruitment via C-X-C motif chemokine ligand 12 (CXCL12) and subsequent thrombospondin 1 (THBS1), production fosters cytotoxic T-cell exhaustion and therapy-resistant microenvironments [53].

In CRC, macrophage infiltration has been correlated with improved suggesting that their functional phenotype—rather than their mere presence—is critical. Macrophage activity is largely determined by the balance between M1 (antitumor) and M2 (protumor) polarization states, shaped by TME-derived signals [54]. In our study, M0 and M1 macrophages were enriched in CRC compared to NCT. Higher M0 and lower M2 levels were associated with improved OS, suggesting that a TME favoring M1-like polarization may support antitumor response. Indeed, cytokines such as IFN-γ, TNF-α, and lipopolysaccharide (LPS) can drive M0 polarization toward M1 phenotype, promoting tumoricidal activity through pro-inflammatory cytokine release.

CRC-derived epidermal growth factor (EGF) promotes M2 polarization via the EGFR/PI3K/AKT/mTOR pathway [54], enhancing immunosuppression and tumor growth. M2 macrophages further sustain CRC development by inducing regulatory T cells (Tregs) through the TGF-β/SMAD pathway. Elevated M2 levels have been linked to poor prognosis, LVI, and resistance to therapies such as oxaliplatin. Targeting macrophage polarization, for example with tasquinimod to shift M2 toward M1 phenotypes, represents a potential strategy to enhance antitumor immunity and limit metastasis [55].

Mast cells also act as independent prognostic factors, with lower densities correlating with better OS. Mast cells promote CRC progression via c-kit/SCF signaling and histamine release [56]. The prognostic impact of intratumoral neutrophils (TANs) remains debated [57], but accumulating evidence links higher intratumoral neutrophil density to malignant phenotypes and poor prognosis [58], consistent with our findings. Overall, our results indicate that the balance between protective immune subsets (plasma cells, naïve B cells, resting CD4^+^ memory T cells, activated dendritic cells, M1-like macrophages) and protumor populations (monocytes, M2 macrophages, mast cells, neutrophils) critically shapes CRC progression and survival, with important implications for immune-based prognostic stratification and therapeutic strategies.

Despite extensive research on CRC genetic drivers, prognosis remains suboptimal, underscoring the need for improved predictive tools. We develop an OS prediction model based on seven prognostic DEGs using Cox survival and LASSO analyses. The model demonstrated strong performance in our primary cohort and was externally validated in TCGA datasets. High-risk patients exhibited significantly poorer survival, with risk scores increasing alongside tumor progression and correlation with advanced pT, pN, pM and stage IV disease.

Among the prognostic genes, ASPH is a transmembrane protein linked to CRC stemness, invasiveness, and drug resistance [59]. It is overexpressed in CRC due to copy number (CN) gain and promoter demethylation [60], accumulates at the invasive tumor front, and promotes progression through Notch downregulation. ASPH silencing or inhibition (SMIs; MO-I-1144) reduces tumor growth and metastasis, highlighting its therapeutic potential [61].

Bradykinin receptors are also implicated in CRC. While BDKRB1 is inducible under inflammatory conditions [62], BDKRB2 (B2R) mediates bradykinin-driven invasion and migration in CRC cells [63]. CACNB1, previously identified in CRC gene expression signatures from GEO and TCGA datasets [64], was more highly expressed in NCT in our cohort and negatively correlated with MYC pathway activation. Notably, CACNB1 agonists may synergize with ERBB2-targeted therapies in ERBB2+ cancers [65].

Chemokines signaling further contributes to tumor behavior. They have mainly antitumor effects, such as X-C motif chemokine receptor 1 (XCR1) and C-X-C motif chemokine receptor 3 (CXCR3) axis, while C-C motif chemokine receptor 4 (CCR4) and C-C motif chemokine receptor 8 (CCR8) usually have pro-tumorigenic responses. While some axes exert antitumor activity, CCR8 activation promotes ERK/MAPK signaling, chemoresistance, EMT, angiogenesis, lymph node metastasis, and Treg recruitment [66]. CCR8-+ Tregs enhance immune suppression and C-C motif chemokine ligand 2 (CCL2)-CCR8 receptor interaction activates TGFβ and nuclear factor kappa B (NF-kB) pathways, supporting proliferation and anti-apoptotic signaling with potential target for drug resistance [67].

Epigenetic regulation also emerged as relevant: CNGA3 hypermethylation and miRNAs mediated repression were correlated with resting CD4^+^ T infiltration in rectal cancer [68]. MAP1A downregulated in our CRC cohort, has been linked to tumor stage and OS in other cancers [69]. OXTR overexpression in CRC correlates with poor OS and immune infiltration (activated CD8^+^ T and B cells); its deletion suppresses proliferation, migration, and invasion, suggesting therapeutic relevance [70].

Overall, genes included in our prognostic model—such as BDKRB1, CACNB1, CNGA3, and MAP1A—are biologically connected to key oncogenic and immune-related pathways. While some have established roles in CRC, others warrant further functional validation to clarify their contribution to tumor progression.

This study has highlighted association of higher risk scores with LVI but not with TILs in CRC patients, suggesting that high-risk score is related with an indicator of advanced stage of CRC. LVI of tumor cells is an early, essential phase for lymphatic metastasis and regulator of tumor immunity. Growing clinical evidence showed that the density of lymphatic vessels within or surrounding growing tumors correlates with poor patient outcome [71]. Additionally, the lymphatic system has been shown to play an important role in tumor immunity, directly inhibiting tumor-specific T cell responses by lymphatic endothelial cells expression of immune checkpoint molecules, such as PD-L1 [72]. Our analysis confirmed the relationship between immune profile, TILs and risk score with the infiltration of pro-inflammatory populations, specifically M1 Macrophages and CD8^+^ T cells, are increased in stage IV vs. III and risk score negatively correlated with M1 Macrophages. Our deconvolution analysis effectively captures the transition toward an “immunologically hot” phenotype. The positive role of TILs in Tumor immune microenvironment (TIME), as the direct physical interaction of TILs with tumor cells involving the physical destruction of them, reduction of the tumor burden, and a longer OS in patients with CRC [73].

Recent studies [74,75] have shown site-specific immune infiltration patterns in CRC along the intestinal tract. In our study, the risk score was strongly associated with a distinct immune profile characterized by reduced resting CD4^+^ T memory cells and dendritic cells, and increased monocytes and neutrophils. However, no significant differences were observed by tumor location (right colon, left colon, or rectum). This suggests that while baseline immune infiltration may vary by site, the 7-gene signature captures a fundamental, site-independent biological axis of tumor progression. The consistent prognostic performance across anatomical subgroups supports its clinical utility as a robust biomarker of disease aggressiveness.

Our results reveal a strong association between immune infiltration patterns and the calculated risk score, indicating a close interplay between tumor-intrinsic molecular alterations and the immune microenvironment. The low-risk group was enriched with resting memory CD4^+^ T cells, M1 macrophages, and activated dendritic cells, reflecting a relatively preserved antitumor immune surveillance state that may help suppress residual tumor expansion and reduce recurrence risk. In contrast, increased monocyte and neutrophil infiltration in the high-risk group suggests the establishment of an immunosuppressive and pro-metastatic microenvironment. Tumor-associated monocytes may promote immune evasion and tumor progression through factors such as THBS1 and TGF-β, while neutrophils can facilitate metastatic dissemination via NET formation. Such immune suppression may impair cytotoxic responses and reduce treatment sensitivity. Collectively, these findings suggest that CRC recurrence and heterogeneous therapeutic responses arise from the interaction between tumor cell–intrinsic proliferative advantages and immune microenvironment remodeling. High-risk molecular signatures may therefore promote therapy-resistant clonal expansion through coordinated cell cycle dysregulation and immune tolerance.

Our seven-gene signature model serves as an independent prognostic factor for OS. By integrating this model with pM, we created nomograms to predict OS in CRC patients. We consider these seven biomarker genes valuable for prognosis prediction and as potential new molecular targets for CRC. Calibration charts confirmed the robustness of these signatures in accurately predicting 1-, 3-, and 5-year survival rates, which aids in systematic planning for patient follow-up.

A key limitation of our study is the relatively small CRC sample size; however, this was offset by deep sequencing (70 million reads), ensuring reliable transcript detection and solid statistical power. The use of matched tumor–normal pairs from the same individuals minimized genetic background noise, while the single-center, standardized NGS workflow reduced technical variability. Moreover, the genetic homogeneity of the Sardinian population enhanced our ability to detect biologically meaningful biomarkers.

The reduced prognostic performance observed in TCGA likely reflects the shift from a genetically isolated population to a highly heterogeneous, multi-ethnic dataset. Although LASSO and AIC-based stepwise selection were applied to limit overfitting, the stronger results in our discovery cohort are plausibly due to reduced biological and technical variability, which is inherently diluted in large multi-institutional datasets. Differences in transcriptomic platforms (microarray vs. non-uniform NGS pipelines) may further explain partial validation in TCGA. In contrast, our Total RNA-seq approach enables broader transcriptomic coverage, including long non-coding RNAs, enabling more comprehensive molecular characterization.

Future studies will focus on expanding primary CRC sample collection across Sardinian centers, enabling independent replication within the same genetic background and the establishment of a unified regional cohort to further validate the clinical utility of the signature.

## 5 Conclusions

Our findings demonstrate the clinical and biological relevance of an integrated prognostic model that captures tumor aggressiveness through combined molecular and immune microenvironment signatures. Low-risk patients showed enrichment of resting CD4 memory T cells, M0 macrophages, and activated dendritic cells, whereas high-risk patients exhibited increased monocyte infiltration. The risk score was positively correlated with neutrophils and monocytes, and negatively correlated with resting CD4 memory T cells, M1 macrophages, and resting/activated dendritic cells, highlighting the role of immune dysregulation in CRC progression. The seven-gene signature functioned as an independent predictor of OS. Integration with pM variables enabled the construction of nomograms for individualized survival prediction. Calibration analyses confirmed high accuracy for 1-, 3- and 5-year survival estimates, supporting the clinical potential of this model for CRC risk stratification and follow-up planning.

## Data Availability

The RNA-seq dataset generated for the study will be made available at European Genome-phenome Archive (EGAC50000000458) upon acceptance of the paper. Please contact the corresponding author for further information if necessary.

## Supplementary Materials

The supplementary material is available online at https://www.techscience.com/doi/10.32604/or.2026.077466/s1.

## Abbreviations

AI	artificial intelligence
AJCC	American joint committee on cancer
APC	APC regulator of Wnt signaling pathway
ASPH	aspartate beta-hydroxylase
ASPM	assembly factor for spindle microtubules
AUC	area under curve
BDKRB1	bradykinin receptor B1
BGN	Biglycan
BK	Bradykinin
BP	biological processes
BRAF	B-Raf murine sarcoma viral oncogene homolog B
BUB1	mitotic checkpoint serine/threonine kinase
BUB1B	mitotic checkpoint serine/threonine kinase B
C-MYC	MYC proto-oncogene, BHLH transcription factor
CACNB1	calcium voltage-gated channel auxiliary subunit beta 1
CC	cellular components
CCL2	C-C motif chemokine ligand 2
CCNA2	cyclin A2
CCNB1	cyclin B1
CCR4	C-C motif chemokine receptor 4
CCR8	C-C motif chemokine receptor 8
CCR8	C-C motif chemokine receptor 8
CD73	ectonucleotidases 73
CDK1	cyclin dependent kinase 1
CDKs	cyclin-dependent kinases
cfDNA	cell free DNA isolation
CIBERSORTx	cell-type identification by estimating relative subsets of RNA transcripts
CIN	chromosomal instability
CMS	molecular subtypes
CN	copy number
CNGA3	cyclic nucleotide gated channel subunit alpha 3
CRC	colorectal cancer
CTNNB1	Catenin Beta 1
CXCL12	C-X-C motif chemokine ligand 12
CXCR3	C-X-C motif chemokine receptor 3
DEGs	differentially expressed genes
DLGAP5	DLG associated protein 5
ECM	extracellular matrix
EGF	epidermal growth factor
EGFR	epidermal growth factor receptor
EMT	epithelial-mesenchymal transition
ERBB2	erb-b2 receptor tyrosine kinase 2
FAP	fibroblast activation protein alpha
FC	fold change
FDR	false discovery rate
FFPE	formalin-fixed, paraffin-embedded
GADD45B	growth arrest and DNA damage inducible beta
GEO	gene expression omnibus
GPCRs	G-protein-coupled receptors
GSEA	gene set enrichment analysis
HG	hub genes
HR	hazard ratio
IFN-γ	interferon gamma
IL-10	interleukin 10
INHBA	inhibin subunit beta A
JAK	Janus kinase
KEGG	Kyoto encyclopedia of genes and genomes
Ki67	marker of proliferation Ki-67
KIF11	kinesin family member 11
KIF20A	kinesin family member 20A
KIF2C	kinesin family member 2C
KIFs	kinesins
KRAS	Kirsten rat sarcoma viral proto-oncogene
LASSO	the least absolute shrinkage and selector operation
LPS	lipopolysaccharide
LVI	lymphovascular invasion
MAP1A	microtubule associated protein 1A
MF	molecular functions
MSI	microsatellite instability
MYBL2	MYB proto-oncogene like 2
NCTs	normal colorectal tissues
NF-kB	nuclear factor kappa B
NGS	next-generation sequencing
NK	natural killer
NRAS	neuroblastoma RAS viral oncogene homolog
OS	overall survival
OXTR	oxytocin receptor
PCA	principal component analysis
PD1	programmed death-1
PNI	perineural invasion
PPI	protein–protein interaction
RNAseq	RNA-sequencing
ROC	receiver operating characteristic analysis
SAF	spindle apparatus formation
SIGLEC5	sialic acid binding Ig like lectin 5
SMIs	small molecule inhibitors
STAT	signal transducer and activator of transcription
STAT3	signal transducer and activator of transcription 3
STRING	search tool for the retrieval of interacting genes/proteins
TAMs	tumor-associated macrophages
TANs	intratumoral neutrophils
TCGA	the cancer genome atlas database
TGF-β	transforming growth factor beta
THBS1	thrombospondin 1
THCs	tumor hybrid cells
TILs	tumor infiltrating lymphocytes
TIME	tumor immune microenvironment
TMB	tumor mutational burden
TME	tumor microenvironment
TNF-α	tumor necrosis factor alfa
TNM	tumor–node–metastasis
TP53	tumor protein P53
Tregs	regulatory T cells
WNT	WNT family member
XCR1	X-C motif chemokine receptor 1
XNA	Xenonucleic acid
