# Supplementary material for: Seven-Gene Signature and Immune Microenvironment as Determinants of Survival Rate in Colorectal Carcinoma
Source: Oncol Res. 2026 Oct 28;34(11):16. doi: 10.32604/or.2026.077466 (PMC13732344; doi:10.32604/or.2026.077466)
Supplement: Supplementary file 1 [file OncolRes-34-77466-s001.zip › Table_S1.docx]

**Table S1.**

Clinical and pathological features of 131 colorectal carcinoma analyzed for transcriptomic profiling.

| **Clinical and pathological features** | | **Number (%)** |
| --- | --- | --- |
| Sex | Male | 78 (59.5) |
|  | Female | 53 (40.5) |
| Age (years) | Median (range) | 69 (33-91) |
| Site | Colon-right | 64 (48.8) |
|  | Colon-left | 36 (27.5) |
|  | Rectum | 30 (22.9) |
|  | *Missing* | 1 (0.8) |
| Histologic grade | G1 | 8 (6.1) |
|  | G2 | 85 (64.9) |
|  | G3 | 30 (22.9) |
|  | G4 | 1 (0.8) |
|  | *Missing* | 7 (5.3) |
| Pathologic tumor size | pT1 | 7 (5.6) |
|  | pT2 | 21 (16.0) |
|  | pT3 | 81 (61.8) |
|  | pT4 | 16 (12.2) |
|  | *Missing* | 6 (4.9) |
| Regional lymph nodes status | pN0 | 56 (42.7) |
|  | pN1 | 40 (30.5) |
|  | pN2 | 26 (19.9) |
|  | *Missing* | 9 (6.9) |
| Metastasis | pM0 | 92 (70.2) |
|  | pM1 | 31 (23.7) |
|  | *Missing* | 8 (6.1) |
| AJCC stage | 0-I | 21 (16.0) |
|  | II | 26 (19.8) |
|  | III | 45 (34.4) |
|  | IV | 31 (23.7) |
|  | *Missing* | 8 (6.1) |
| TILs score | High | 13 (9.9) |
|  | Low | 86 (65.6) |
|  | *Missing* | 32 (24.5) |
| LVI | Absent | 74 (56.5) |
|  | Present | 32 (24.4) |
|  | *Missing* | 25 (19.1) |
| PNI | Absent | 83 (63.4) |
|  | Present | 24 (18.3) |
|  | *Missing* | 24 (18.3) |

*Grade: G1 Well differentiated, G2 Moderately differentiated, G3 Poorly differentiated; AJCC stage according to WHO criteria (Hamilton SR, 2010); TILs: Tumor infiltrating lymphocytes; LIV: Lympho-vascular invasion; PNI: Perineural invasion.*
