# Supplementary material for: Seven-Gene Signature and Immune Microenvironment as Determinants of Survival Rate in Colorectal Carcinoma
Source: Oncol Res. 2026 Oct 28;34(11):16. doi: 10.32604/or.2026.077466 (PMC13732344; doi:10.32604/or.2026.077466)
Supplement: Supplementary file 1 [file OncolRes-34-77466-s001.zip › Table_S5.docx]

| **Table S5**. CIBeRSORT fraction comparison of 22 infiltrating immune cells in colorectal carcinoma (CRC) and normal colon tissues (NCT). | | | | | |
| --- | --- | --- | --- | --- | --- |
| **Immune cell type** | **Most represented in** | **P.value** | **P.adj Bonferroni** | **effect size metrics** | **Impact effect** |
| Mast.cells.resting | NCT | 1.58e-19 | 3.48e-18 | 0.798883 | large |
| Macrophages.M0 | CRC | 3.33e-18 | 7.33e-17 | 0.770115 | large |
| Mast.cells.activated | CRC | 8.44e-15 | 1.86e-13 | 0.705545 | large |
| NK.cells.resting | CRC | 3.52e-13 | 7.74e-12 | 0.640449 | large |
| Macrophages.M2 | NCT | 8.43e-12 | 1.85e-10 | 0.60154 | large |
| Plasma.cells | NCT | 2.89e-09 | 6.36e-08 | 0.522894 | large |
| Dendritic.cells.activated | CRC | 1.39e-08 | 3.06e-07 | 0.499612 | moderate |
| T.cells.CD4.memory.activated | CRC | 5.15e-08 | 1.13e-06 | 0.506776 | large |
| NK.cells.activated | NCT | 1.2e-07 | 2.64e-06 | 0.50921 | large |
| Macrophages.M1 | CRC | 1.9e-06 | 4.18e-05 | 0.412382 | moderate |
| T.cells.CD4.naive | CRC | 1.06e-05 | 0.000233 | 0.409217 | moderate |
| B.cells.memory | NCT | 1.14e-05 | 0.000251 | 0.373202 | moderate |
| T.cells.CD8 | NCT | 1.39e-05 | 0.000306 | 0.3943 | moderate |
| Neutrophils | CRC | 2.14e-05 | 0.000471 | 0.417802 | moderate |
| Eosinophils | CRC | 8.31e-05 | 0.001828 | 0.33383 | moderate |
| B.cells.naive | NCT | 0.0256 | 0.5632 | 0.198584 | small |
| T.cells.CD4.memory.resting | NCT | 0.123 | >0.99 | 0.135872 | small |
| T.cells.follicular.helper | CRC | 0.176 | >0.99 | 0.086039 | small |
| T.cells.regulatory..Tregs. | NCT | 0.478 | >0.99 | 0.110987 | small |
| T.cells.gamma.delta | NCT | 0.295 | >0.99 | 0.073018 | small |
| Monocytes | NCT | 0.318 | >0.99 | 0.08796 | small |
| Dendritic.cells.resting | CRC | 0.2 | >0.99 | 0.105789 | small |

The Wilcoxon signed rank outcomes and the impact of each cell subtype on colorectal carcinoma and normal colon tissues. Statistical analysis was conducted as described in the Material and methods section using rstatix R package.
