# Supplementary material for: Seven-Gene Signature and Immune Microenvironment as Determinants of Survival Rate in Colorectal Carcinoma
Source: Oncol Res. 2026 Oct 28;34(11):16. doi: 10.32604/or.2026.077466 (PMC13732344; doi:10.32604/or.2026.077466)
Supplement: Supplementary file 1 [file OncolRes-34-77466-s001.zip › TSP_OR_77466-Fig._S1.pdf]

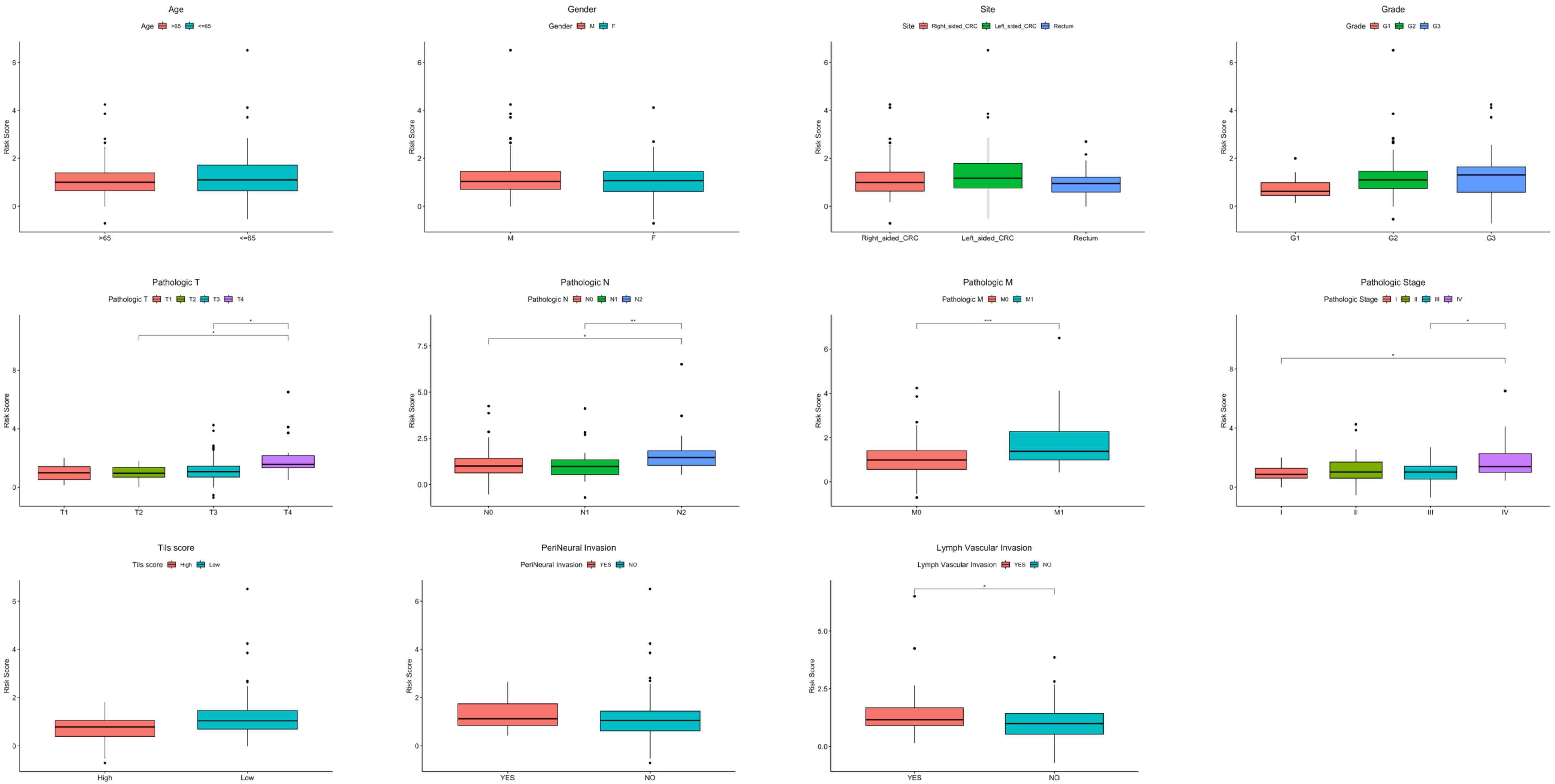

Figure S1. Risk scores distribution in different clinical and pathological features.
Box plots of CRC clinical pathological features. *$P < 0.05$, **$P < 0.01$, ***$P < 0.001$; Kruskal-Wallis test followed by Dunn's multiple comparison test.
