# Supplementary material for: Seven-Gene Signature and Immune Microenvironment as Determinants of Survival Rate in Colorectal Carcinoma
Source: Oncol Res. 2026 Oct 28;34(11):16. doi: 10.32604/or.2026.077466 (PMC13732344; doi:10.32604/or.2026.077466)
Supplement: Supplementary file 1 [file OncolRes-34-77466-s001.zip › TSP_OR_77466-Fig._S2.pdf]

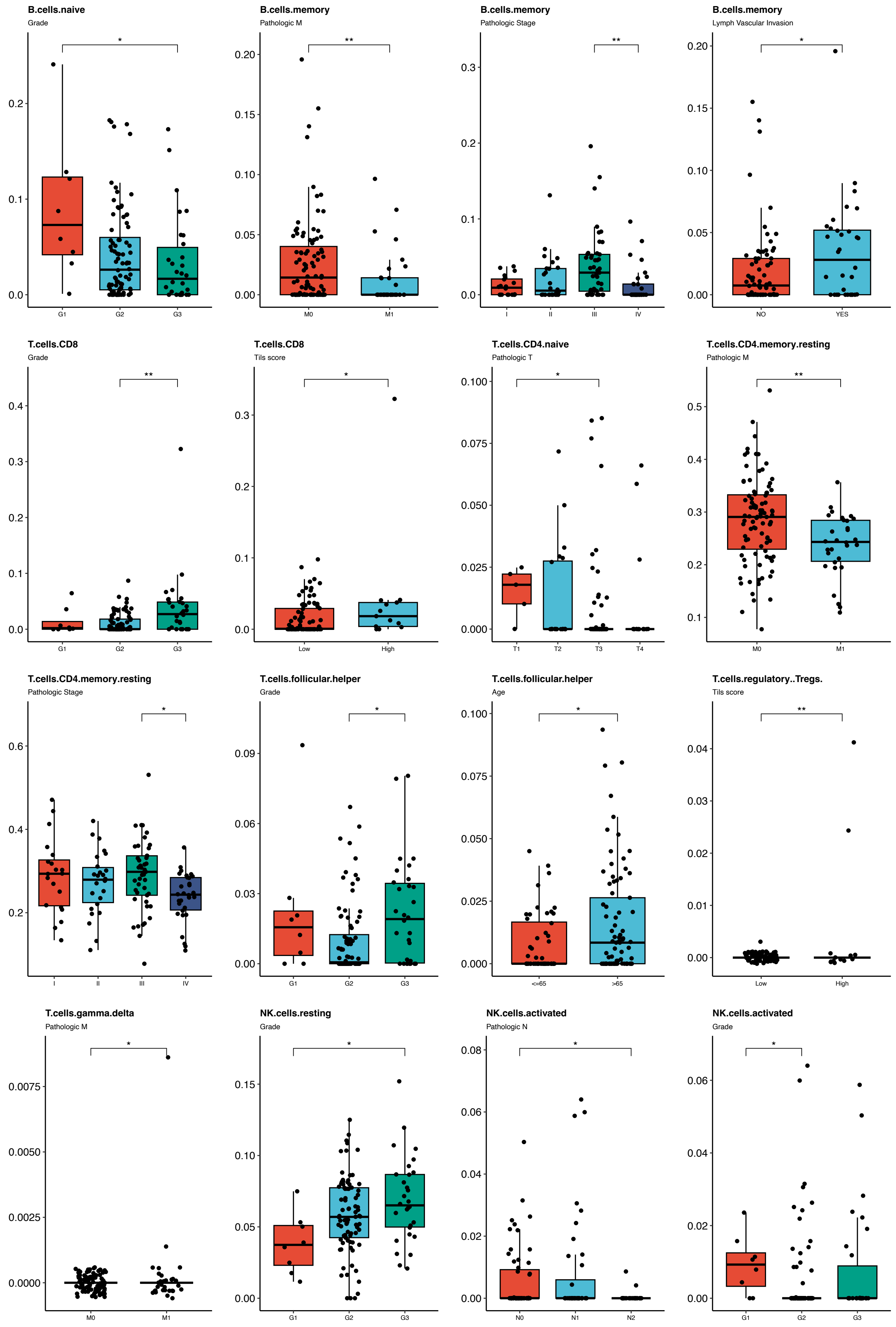

Figure S2. Relationship between immune profile and clinical features.
Box plots of immune cells within CRC clinical pathological features. **P*< 0.05, ***P*< 0.01, ****P*< 0.001; Kruskal-Wallis test followed by Dunn's multiple comparison test.
